# A gain-of-function mouse model identifies PRMT6 as a NF-κB coactivator

**DOI:** 10.1093/nar/gku530

**Published:** 2014-06-17

**Authors:** Alessandra Di Lorenzo, Yanzhong Yang, Marc Macaluso, Mark T. Bedford

**Affiliations:** The University of Texas MD Anderson Cancer Center, Science Park, P.O. Box 389, Smithville, TX 78957, USA

## Abstract

Protein arginine methyltransferase 6 (PRMT6) is a nuclear enzyme that modifies histone tails. To help elucidate the biological function of PRMT6 *in vivo*, we generated transgenic mice that ubiquitously express PRMT6 fused to the hormone-binding portion of the estrogen receptor (ER*). The ER*-PRMT6 fusion is unstable and cytoplasmic, but upon systemic treatment with tamoxifen, it becomes stabilized and translocates into the nucleus. As a result, a dramatic increase in the H3R2me2a histone mark is observed. We found that one consequence of induced ER*-PRMT6 activation is increased IL-6 levels. IL-6 expression is regulated by the nuclear factor-kappa B (NF-κB) transcription factor, and PRMT6 functions as a coactivator of this pathway. We show that PRMT6 directly interacts with RelA, and that its overexpression enhances the transcriptional activity of an ectopic NF-κB reporter and endogenously regulates NF-κB target genes. PRMT6 is recruited, by RelA, to selective NF-κB target promoters upon TNF-α stimulation. Moreover, ER*-PRMT6 activation causes RelA accumulation in the nucleus. In summary, we observe that PRMT6 is recruited to chromatin at selective NF-κB target promoters, where it likely impacts the histone code and/or methylates other chromatin-associated proteins to facilitate transcription.

## INTRODUCTION

Arginine methylation is a prevalent posttranslational modification found on both nuclear and cytoplasmic proteins. The methylation of arginine residues is catalyzed by the protein arginine *N-*methyltransferase (PRMT) family of enzymes (1,2). The complexity of the methylarginine mark is enhanced by the ability of this residue to be methylated in three different fashions on the guanidino group: monomethylated (MMA), symmetrically dimethylated (SDMA) and asymmetrically dimethylated (ADMA), with the potential of different functional consequences for each methylated state. There are nine PRMTs: type I and type II enzymes catalyze the formation of a MMA intermediate, then type I PRMTs (PRMT1, 2, 3, 4, 6 and 8) further catalyze the production of ADMA, while the type II PRMT (PRMT5) catalyzes the formation of SDMA. PRMT7 appears to be a type III enzyme that catalyzes only MMA formation; PRMT9 has yet to be characterized.

PRMT6 is a nuclear enzyme (3), which methylates both histone and non-histone proteins (4–6). PRMT6 feeds into the epigenetic code and possesses both transcriptional repressor and activator activities. It is the primary enzyme responsible for H3R2 methylation in mammalian cells (7–9). The H3R2me2a mark is thought to be repressive in nature because of its ability to counteract the activator function of the H3K4me3 mark by inhibiting effector protein recruitment. PRMT6 also deposits the H2AR29me2a mark, which is enriched at the promoters of genes that are transcriptionally repressed by PRMT6 (10). With regard to its activator functions, PRMT6 was identified as a transcriptional coactivator of the estrogen receptor (11), and in this context, its ability to methylate H3R42 may be important. The H3R42 is a non-tail histone methylation event that positively affects transcription, likely by disrupting the histone–DNA interaction and making the nucleosome slidable (12). In addition, PRMT6 can methylate H4R3 and/or H2AR3 *in vitro*, which may correlate with actively transcribed loci (13). It is, thus, clear that PRMT6 modifies a number of sites on histones, but it is not obvious how it changes its specificity to toggle between a coactivator and a corepressor.

As a transcriptional repressor, PRMT6 suppresses the expression of thrombospondin-1, which is a potent natural inhibitor of angiogenesis and endothelial cell migration (14). In addition, the cyclin-dependent kinase inhibitor, p21, is also transcriptionally repressed by PRMT6 (15–17). The PRMT6-knockout (KO) mouse is viable, but mouse embryonic fibroblasts (MEFs) from null embryos undergo rapid cellular senescence (16). The reason for this senescence phenotype is the release of p53 and p21 repression with PRMT6 loss. It is, thus, likely that in tumors with high PRMT6 levels, the p53 pathway is epigenetically silenced. In keeping with this hypothesis, PRMT6 has been reported to be overexpressed in bladder and lung cancer cells (18), and more recently also been found to be dramatically elevated in prostate carcinomas (19).

In the current study, we identified PRMT6 as a coactivator for nuclear factor-kappa B (NF-κB). Three other PRMTs have previously been implicated in the regulation of the NF-κB pathway. Indeed, CARM1 was shown to function as a promoter-specific coactivator for NF-κB in concert with p300/CBP and SRC-2/GRIP1 through deposition of the H3R17me2a active mark at NF-κB-regulated promoters (20,21). PRMT1 was reported to coactivate NF-κB-dependent gene expression synergistically with CARM1 and PARP1 and its methyltransferase activity was required for the coactivation (22). Furthermore, RelA is a substrate for PRMT5, which also activates NF-κB (23). Here we found that transgenic mice induced to ubiquitously overexpress PRMT6 display increased production of interleukin 6 (IL-6), a pleiotropic cytokine regulated by NF-κB. Further investigation revealed that PRMT6 binds directly to RelA and that it functions as a coactivator for a number of NF-κB target genes.

## MATERIALS AND METHODS

### Antibodies, cells and reagents

HEK 293 and HeLa cells were purchased from ATCC and cultured in Dulbecco's modified Eagle medium (DMEM) containing pen-strep, non-essential amino acids and Fetal Bovine Serum (FBS) 10%. Tamoxifen (Tamox) (cat. # T5648), 4-hydroxytamoxifen (OHT) (cat. # H7904) and tumor necrosis factor alpha (TNF-α) (cat. # T6674) were purchased from Sigma. Anti-RelA antibody for western blotting (WB), chromatin immunoprecipitation (ChIP) and immunofluorescence (IF) was from Abcam (cat. # ab16502). Anti-PRMT6 antibody for WB and ChIP (on human cells) was from Imgenex (cat. # IMG-506). Anti-PRMT6 antibodies for immunoprecipitation (IP) and WB on mouse cells were from Bethyl Laboratories (cat. # A300-928A and A300-929A). Anti-Flag antibody (cat. # F3165) and anti-Flag agarose (cat. # A2220) were obtained from Sigma. Anti-H3R2me2a antibody was from Millipore (cat. # 05-808). Anti-GFP antibody was from Life Technologies (cat. # A6455). Anti-Lamin A/C antibody was from Santa Cruz (cat. # sc-20681). Protein A/G Ultra Link resin was from Thermo Scientific (cat. # 53132).

### Transgene construction and mouse generation

Estrogen receptor hormone-binding domain variant (ER*) that binds Tamox, but not estrogen, was a gift from Martin McMahon (24). ER* was generated by polymerase chain reaction (PCR) (from the pBP3hbER* vector) and cloned into a pCAGGS construct containing flagged human PRMT6 cDNA. An aliquot of the construct produced by Midi Prep using Qiagen kit (Qiagen Scientifics, MD, USA) was linearized overnight using ScaI, and then column purified using PCR purification kit from Qiagen (Qiagen Scientifics, MD, USA). This transgene was injected into the male pronucleus of day 1-fertilized (FVB) embryos. Injected embryos were transferred into day 1-plugged pseudo-pregnant foster mice, and litters were screened for the presence of the transgene using Southern blot analysis to identify heterozygous founders. Founder mice were backcrossed to establish lines of animals.

### Southern blot genotyping

Approximately 1000 bp of PRMT6 cDNA sequence was cloned out from a pCAGGS-Flag-PRMT6 construct to use as a template for probe generation by PCR using the primers For. 5′-ATGGACTACAAGGACGACGATGACAA-3′ and Rev. 5′-GATAGGCAGCCTGCACCTGAGGAGTG-3′. The radiolabeled hybridization probe was prepared with ^32^P-dCTP using the Random Primer Labeling kit from Agilent Technologies (cat. # 300385), following manufacturer's instructions. Genomic DNA was isolated from the tails of 3-week-old mice and digested EcoRI enzyme. Digested DNA was run on 1% agarose gel overnight and transferred to a nitrocellulose membrane. The membrane was then cross-linked and incubated in 10-ml Southern hybridization buffer containing sodium dodecyl sulphate (SDS), the radioactive probe and salmon sperm DNA overnight at 42°C, then washed with saline-sodium citrate (SSC) buffer (3.0 mol /l NaCl, 0.3 mol/l sodium citrate) and exposed to film for 1–3 days at −80ºC.

### Administration of Tamox to mice

To activate ER*-PRMT6 in adult transgenic mice by Tamox topical administration, Tamox was dissolved in ethanol (1 mg/200 μl) and applied topically to a shaved area of dorsal skin. As control, wild-type (WT) littermates treated with Tamox or transgenic littermates treated with ethanol, were included in these studies. For intraperitoneal injections, Tamox (1 mg/100 μl) was dissolved in sunflower oil.

### Stable cell line generation

HEK 293 cells were transfected with the pCAGGS-ER*-PRMT6 construct (10 μg) along with an empty plasmid carrying neomycin resistance (1 μg). Selection of stable transfected cells was performed using neomycin (G418).

### Immunostaining of mouse tissue and immunofluorescence on cultured cells

Tissue samples were obtained from WT or transgenic mice, Tamox or vehicle treated. Tissues were fixed overnight in neutral buffered formalin (24 h), transferred to 70% ethanol, then embedded in paraffin and sectioned (5 μm). Slides were de-paraffinized with standard procedure. For imm try, non-specific antibody binding was blocked by incubating slides with Biocare Blocking Reagent (cat# BS966M) for 10 min. Slides were drained and incubated with primary anti-Flag antibody for 30 min at room temperature, then washed with phosphate buffered saline (PBS) and incubated with biotinylated rabbit antimouse secondary antibody for 15 min at room temperature. After washing, slides were incubated with SA-HRP (Biocare) for 30 min at room temperature, washed again and incubated with BioGenex DAB monitoring staining development. Slides were then washed, counterstained, dehydrated and mounted. For immunofluorescence (IF), slides were deparaffinization and then blocked with 20% FBS 30 min, washed and incubated with primary antibody for 1 h, then with secondary antibody Texas Red-conjugated, followed by 4',6-diamidino-2-phenylindole (DAPI) staining. In the case of cultured cells, cells were grown on a coverslip. At the time of IF, cells were rinsed with PBS and fixed with 4% paraformaldehyde for 15 min at room temperature. Cells were then washed with washing solution (0.1% NP-40 in PBS), blocked in 20% FBS and incubated with primary antibody anti-RelA (in washing solution) 1 h at room temperature. Cells were then washed, incubated with secondary antibody (Alexa Fluor 555-conjugated) for 30 min, washed again and DAPI-stained afterwards.

### Isolation of mouse embryonic fibroblasts

Briefly, a 12.5-day-plugged mouse was sacrificed following standard procedure. Intact embryos were collected and placed in six-well plates containing 3 ml DMEM, 10% FBS and pen-strep. Embryos were mechanically disrupted passing them through a 5-ml syringe with a 18G needle five times, and then plated in 10-cm dish containing 10 ml of media. Media were changed every day for 3 days.

### Western blotting and coimmunoprecipitation

For total cell lysates, cultured cells (80% confluency) were scraped of a petri dish, lysed in RIPA buffer containing protease inhibitor cocktail and denatured by boiling. In the case of cell fractionation, the NE-PER kit from Thermo Scientific (cat. # 78835) was employed. For tissue lysates, tissues were harvested, snap frozen in liquid nitrogen, grounded using a mortar, transferred in RIPA buffer with protease inhibitors and sonicated three times using Fisher Scientific Sonicator Model 500 (25% power, 10 s each cycle). Protein lysates (30–50 μg) were resolved on sodium dodecyl sulphate-polyacrylamide gel electrophoresis (SDS-PAGE) gel. For the coimmunoprecipitations (co-IPs), cells were lysed in a mild co-IP buffer [50 mM Tris–HCl (pH 7.5), 150 mM NaCl, 0.1% Nonidet P-40, 5 mM ethylenediaminetetraacetic acid (EDTA), 5 mM ethylene glycol tetraacetic acid (EGTA), 15 mM MgCl_2_] containing protease inhibitor cocktail and incubated with appropriate primary antibody overnight at 4ºC. Lysates were then incubated with protein A/G agarose the next day for 3 h at 4ºC, the agarose washed in co-IP buffer three times, denatured by boiling and resolved on SDS-PAGE gel. When using anti-Flag agarose, we followed the same procedure as with protein A/G agarose. The resolved proteins were transferred on polyvinylidene difluoride (PVDF) membranes, incubated in 5% non-fat milk for 30 min at room temperature and subsequently incubated with specific primary antibody in non-fat milk overnight at 4ºC. The blots were washed three times in PBS containing 0.2% Tween-20 and incubated for 1 h in peroxidase-conjugated IgG diluted 1/5000 in non-fat milk at room temperature. Blots were washed and immune complexes detected with enhanced chemiluminescence (ECL).

### Acid extraction of histones

For histone purification, cells were first fractionated using NE-PER kit from Thermo Scientific (cat. # 78835) according to the manufacturer's protocol. After removing the nuclear fraction, 150 μl of 0.8-M HCl was added to the chromatin pellet (15 μl) and sonication at 30% power using Fisher Scientific Sonicator Model 500 (15 s) was performed. Samples were then centrifuged (13 000 rpm, 10 min) and the supernatant was neutralized (to pH = 7.00) by adding Tris–HCl 1.5 M, pH 8.5 (90 μl) and loaded on SDS-PAGE gel for quantification by coomassie staining (5 μl).

### Chromatin immunoprecipitation

Cells at 80% confluency were treated with TNF-α or PBS (vehicle). ChIP analysis was performed using the Millipore Magna ChIP assay kit protocol (cat# 17-610). For the immunoprecipitation, 2 μg of appropriate antibody was used for each condition and incubated overnight at 4ºC, with lysates from 10^6^ cells. Quantitative real-time PCR (RT-PCR) was performed with the Applied Biosystems 7900HT RT-PCR instrument using the iTaq Universal SYBR Green Supermix form Bio-Rad (cat. # 172-5121).

The primer sequences were as follows:

Intergenic region 8000 upstream IL-6 promoter:

For. 5′- GCTCCTCCATCTGGTGTCAT-3′, Rev.5′-AAATTGGGGGTAGGGTTGTC-3′

IL-6 TSS:

For. 5′-AATGTGGGATTTTCCCATGA-3′, Rev. 5′-AGTTCATAGCTGGGCTCCTG-3′

TNF-α TSS:

For. 5′-ATCGGAGCAGGGAGGATG-3′, Rev. 5′-CCAGCGGAAAACTTCCTT-3′

IκB-α TSS:

For. 5′-GGAAGGACTTTCCAGCCACT-3′, Rev. 5′-GGAATTTCCAAGCCAGTCAG-3′

For the ChIP/re-ChIP experiment, the immunoprecipitated DNA–protein complex was eluted from the beads by incubating with 50 µl of 10-mM 10mM Dithiothreitol (DTT) diluted in Tris-EDTA (TE) buffer at 37ºC for 30 min. The eluted supernatant was diluted 20 times in ChIP dilution buffer from the Millipore Magna ChIP assay kit, and second round of ChIP was performed using the αPRMT6 antibody.

### RNA isolation and quantitative RT-PCR

RNA was isolated from 80% confluent cell plates using TRIzol Reagent (Invitrogen, Carlsbad, CA, USA). cDNA was prepared from total RNA (1 μg) using the Superscript III First Strand Synthesis System for RT-PCR from Invitrogen (cat. # 18080-051) following the manufacturer's protocol. Quantitative RT-PCR was performed with the Applied Biosystems 7900HT RT-PCR instrument using the iTaq Universal SYBR Green Supermix form Bio-Rad (cat. # 172-5121) with primers for the indicated genes. Primers were designed across exons.

The primer sequences were as follows:

IL-6:

For. 5′-ACTCACCTCTTCAGAACGAATTG-3′, Rev.5′-CCATCTTTGGAAGGTTCAGGTTG-3′

TNF-α:

For. 5′-CCCCAGGGACCTCTCTCTAA-3′, Rev. 5′-TGAGGTACAGGCCCTCTGAT-3′

MCP1/CCL2:

For. 5′-TCTGTGCCTGCTGCTCATAG-3′, Rev. 5′-GCTTCTTTGGGACACTTGCT-3′

COX-2:

For. 5′- TGAAACCCACTCCAAACACA-3′, Rev. 5′-GAGAAGGCTTCCCAGCTTTT-3′

IKB-α (nuclear factor of kappa light polypeptide gene enhancer in B-cells inhibitor, alpha):

For. 5′-AGACCTGGCCTTCCTCAACT-3′, Rev. 5′-TGCTCACAGGCAAGGTGTAG-3′

GAPDH (Glyceraldehyde 3-phosphate dehydrogenase):

For. 5′-AGCCACATCGCTCAGACAC-3′, Rev. 5′-GCCCAATACGACCAAATCC-3′

### Luciferase reporter assay

HEK 293 cells were cultured in 10% FBS-supplemented DMEM in 24-well plates to 70% confluency. For reporter gene assays, cells were cotransfected with 200 ng of the NF-κB luciferase reporter plasmid, 25 ng Renilla control luciferase plasmid and the indicated constructs (400 ng). The total plasmid content was balanced up to 625 ng with empty vectors when necessary. Cells were treated with TNF-α (10 ng/ml) or PBS for 6 h before luciferase assay was done using Dual-Luciferase Reporter Gene Assay from Promega (Madison, WI, USA).

### *In vitro* binding assay

Glutathione S-transferase (GST), GST-RelA (1–431), His-Tudor (UHRF1) and His-PRMT6 were isolated from *Escherichia coli* BL21 cells following 5-h induction with Isopropyl β-D-1-thiogalactopyranoside (IPTG). For GST fusion proteins, cells were harvested in PBS buffer plus protease inhibitors, sonicated and centrifuged to remove cell debris. GST fusion proteins were purified by incubation with glutathione beads (Amersham Biosciences) overnight with rotation at 4ºC. Beads were washed five times and bound proteins were eluted from the beads using freshly prepared reduced glutathione (33 mM). For His-tagged proteins, cells were lysed in appropriate lysis buffer (containing 1 mM EDTA, 1 mM EGTA, 5 mM DTT and protease inhibitors), incubated with Ni-NTA agarose (Qiagen Scientifics, MD, USA) overnight with rotation at 4ºC and then eluted with elution buffer (containing 250 mM imidazole). Ten microgram of eluted GST fusion proteins and His-PRMT6 were incubated in co-IP buffer [50 mM Tris–HCl (pH 7.5), 150 mM NaCl, 0.1% Nonidet P-40, 5 mM EDTA, 5 mM EGTA, 15 mM MgCl_2_] overnight at 4ºC. Complexes were then pulled down with glutathione beads for 2 h at 4°C, washed extensively in co-IP buffer and resolved on SDS-PAGE gel followed by WB analysis.

### Statistical analysis

Statistical analysis was performed using Student's *t*-test. * indicates *P* < 0.05; ** indicates *P* < 0.01; *** indicates *P* < 0.001.

## RESULTS

### Characterization of Tamox-inducible ER*-PRMT6 chimera in cell lines

**Figure 1.**
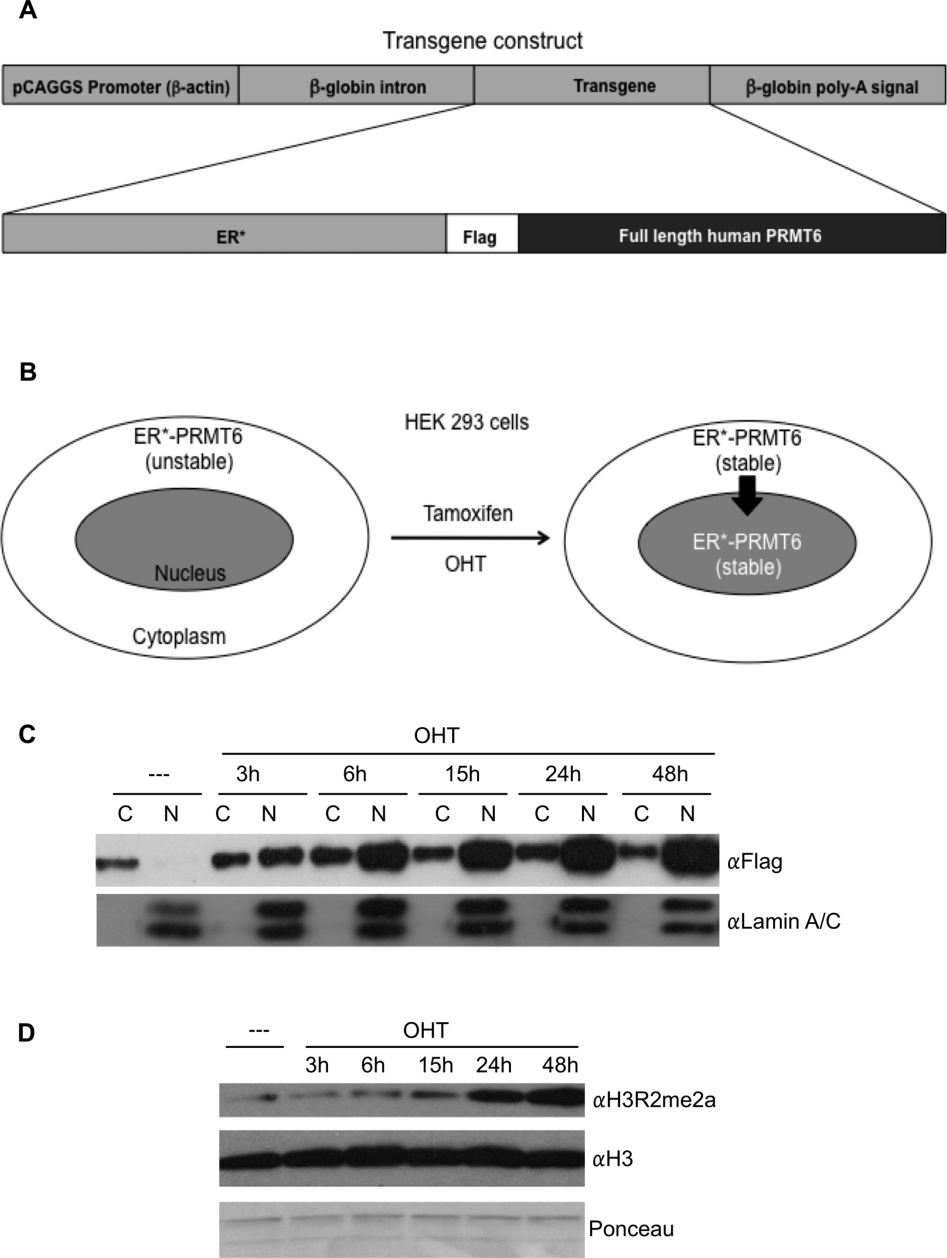
Characterization of an inducible ER*-PRMT6 fusion. (**A**) Human PRMT6 cDNA was cloned into the pCAGGS vector, downstream an ER* (a truncated version of the estrogen receptor that binds Tamox). A Flag-tag was introduced between the two proteins. The ubiquitous β-actin promoter drives the expression of the chimeric ER*-PRMT6 protein. (**B**) Graphic depiction of this approach. ER*-PRMT6 is localized in the cytoplasm. Upon Tamox or OHT treatment, the chimera protein becomes stabilized and translocates into the nucleus. (**C**) HEK293 cells stably transfected with pCAGGS-ER*-PRMT6 were treated with OHT (2 μM) and then separated into nuclear [N] and cytoplasmic [C] fractions. Western analysis was performed using an αFlag antibody to detect ER*-PRMT6. An αLamin A/C Western was performed to confirm the quality of the nuclear/cytoplasmic fractionation. Time points after OHT treatment are indicated. (**D**) Core histones were isolated from the same ER*-PRMT6 HEK 293 cells shown in (C). The core histones were subjected to western analysis with an αH3R2me2a antibody to monitor accumulation of this mark. Equal loading was confirmed by Ponceau staining and αH3 western analysis.

The hormone-binding domain of steroid receptors can be used as a regulatory system to probe protein function (25). This approach has been used successfully to generate conditional forms of transcription factors (c-Myc, Stat3, p53), kinases (c-Abl & Raf1), DNA methyltransferase (MGMT) and Cre recombinase (26,27). The development of a mutant estrogen receptor HBD (ER*) that is unable to bind estrogen, yet retains normal affinity for the synthetic ligand, Tamox or OHT, has enhanced this approach (28). Human PRMT6 was flag-tagged and fused to ER*, and then cloned into the pCAGGS expression vector (Figure 1A). In this system, ER*-PRMT6 expression is driven by the ubiquitous β-actin promoter (29). To test the approach, this expression vector was stably transfected into HEK 293 cells. In the absence of synthetic ligand, ER*-PRMT6 is localized to the cytoplasm; upon Tamox or OHT treatment, the chimeric protein no longer interacts with the hsp90 complex, and is released for translocation into the nucleus where it is stabilized and active (Figure 1B). This is indeed what we observed (Figure 1C). ER*-PRMT6 stable HEK 293 cells were fractionated into nuclear and cytoplasmic parts and subjected to western blot analysis using an αFlag antibody. Prior to OHT treatment, ER*-PRMT6 is restricted to the cytoplasmic fraction. After OHT treatment, ER*-PRMT6 translocates to the nucleus and steadily accumulates there. Since PRMT6 is known to deposit the H3R2me2a mark (7–9), we isolated core histones from the same cells used for the fractionation study in Figure 1C, and performed a western blot analysis with an αH3R2me2a antibody. Within 2 days, the H3R2 site becomes heavily modified (Figure 1D).

### Characterization of Tamox-inducible ER*-PRMT6 transgenic mouse lines

**Figure 2.**
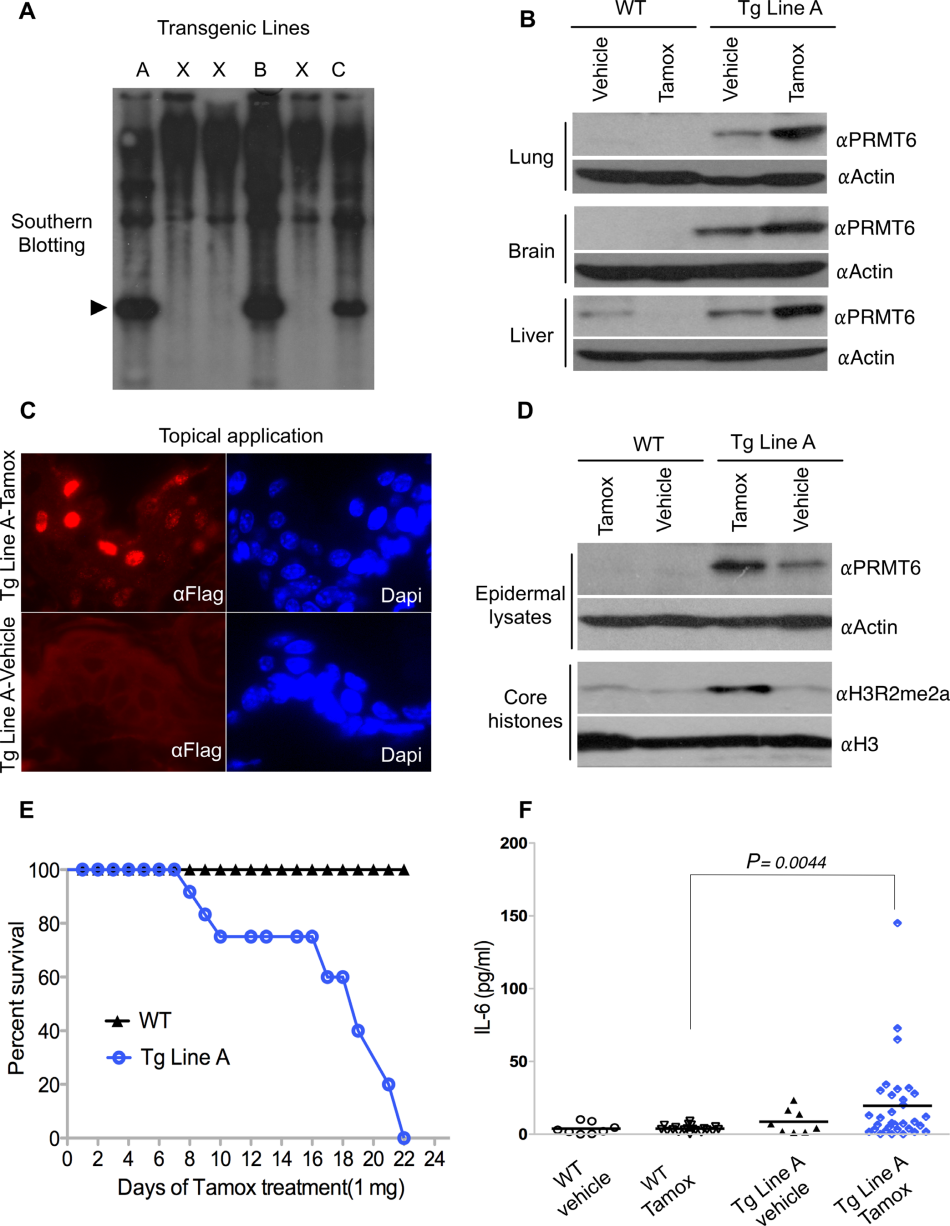
The inducible ER*-PRMT6 fusion is functional *in vivo*. (**A**) Three transgenic lines were established using the pCAGGS-ER*-PRMT6 vector. Southern analysis was performed with a PRMT6 cDNA probe. The arrowhead indicates the transgene. (**B**) Intraperitoneal injection of 1-mg Tamox, for 5 consecutive days, induced stabilization of ER*-PRMT6 in lung, brain and liver of Line A mice. The vehicle (sunflower oil) was injected as a control. (**C**) IF for the Flag-tag performed on skin sections from transgenic Line A mice shows ER*-PRMT6 stabilization in the nucleus when Tamox was applied topically on shaved dorsal skin (1 mg/day) for 5 days. (**D**) Core histones were isolated from epidermal scrapes of topically Tamox-treated mice (1 mg every other day for 10 weeks) and subjected to WB with an αH3R2me2a antibody. (**E**) The ER*-PRMT6 Line A mice die upon prolonged Tamox administration. A survival curve was generated after topical Tamox administration (1 mg/day) until death of all transgenic mice occurred (*n* = 14). WT mice were not impacted by this treatment. (**F**) IL-6 serum levels after 12 topical applications of Tamox (1 mg/day) were measured by ELISA in four groups of animals (WT, WT Tamox-treated, Transgenic (Tg) Line A, Tg Line A Tamox-treated). Wilcox and Student *t*-tests were run and a *P* value of 0.004 was obtained for the Tg Tamox-treated group (*n* = 32) when compared with the WT Tamox-treated group (*n* = 20) for both tests. All the mice in this study were healthy at the time of serum harvesting. All mice were between 8 and 9 weeks of age, and females and males were equally represented.

The pCAGGS-ER*-PRMT6 construct described above was used to generate three founder transgenic mouse lines—A, B and C (Figure 2A). Lines A and C underwent germ-line transmission, but Line C displayed low levels of transgene expression. Subsequent studies were, thus, focused on transgenic Line A. Tamox was administered to Line A mice by daily intraperitoneal injections, for 5 days, as previously described (30). At this point, we analyzed the expression levels of the ER*-PRMT6 chimera in lysates generated from a number of organs (Figure 2B). In addition, immunohistochemical analysis of ER*-PRMT6 localization in the liver shows that intraperitoneal administration of Tamox causes translocation and accumulation of this chimeric protein in the nucleus (Supplementary Figure S1). Apart from intraperitoneal injection, Tamox can also be administered to the surface of the skin from where it is absorbed for the activation of ER* fusion proteins in the epidermis (31), but this delivery method also allows systemic uptake of Tamox. To determine whether ER*-PRMT6 could be induced with topical Tamox treatment, Line A transgenic mice were shaved dorsally and painted with Tamox (1 mg) or vehicle (ethanol) daily for 5 days. We then performed IF using the αFlag antibody on paraffin-embedded skin sections and found that ER*-PRMT6 is strongly induced and localized to the nucleus of epidermal cells in these mice (Figure 2C). Epidermal scrapes were also collected from the Tamox-treated transgenic mice along with controls, and divided in equal amounts for tissue lysates and core histone extraction in order to perform αPRMT6 and αH3R2me2a western analysis, respectively (Figure 2D). This experiment revealed that topical Tamox treatment of transgenic Line A mice results in a marked increase in H3R2me2a levels on the histone tail as a consequence of ER*-PRMT6 nuclear stabilization in the epidermis.

Unexpectedly, ER*-PRMT6 transgenic mice that were subjected to daily topical Tamox treatment all died within a 3-week period (Figure 2E). Death occurred more rapidly (within 12 days) if Tamox (1 mg) was administered via intraperitoneal injections (data not shown). Lethality could be avoided by applying Tamox (1 mg) topically every second day, although we did not observe a strong stabilization of the chimera in internal organs with this treatment (data not shown). In an effort to determine the cause of death of these mice, we performed several serum analyses, including evaluation of inflammatory cytokines such as IL-6, TNF-α and IL-1β, after topical application of Tamox (1 mg) for 12 consecutive days. IL-6 serum levels were significantly elevated in the transgenic Tamox-treated group (Figure 2F). The high variability observed in this assay may be due to the anti-inflammatory effects of Tamox, which has been shown to specifically interfere with NF-κB activation (32–34). No significant difference in TNF-α or IL-1β was detected (data not shown). Since mouse keratinocytes are known to be a good source of IL-6 (35), we sought to confirm that ER*-PRMT6 stabilization increased IL-6 transcription in the Tg Line A primary keratinocytes. Therefore, we isolated these cells from newborn WT and Tg mice (Supplementary Figure S2A), cultured for 1 day, treated with OHT for 2 days (without replacing the media) and stimulated with TNF-α for 60 min prior to extracting RNA. As shown in Supplementary Figure S2B, the keratinocytes derived from transgenic mice also show a significant increase in IL-6 transcription.

### PRMT6 interacts directly with the RelA subunit of NF-κB

**Figure 3.**
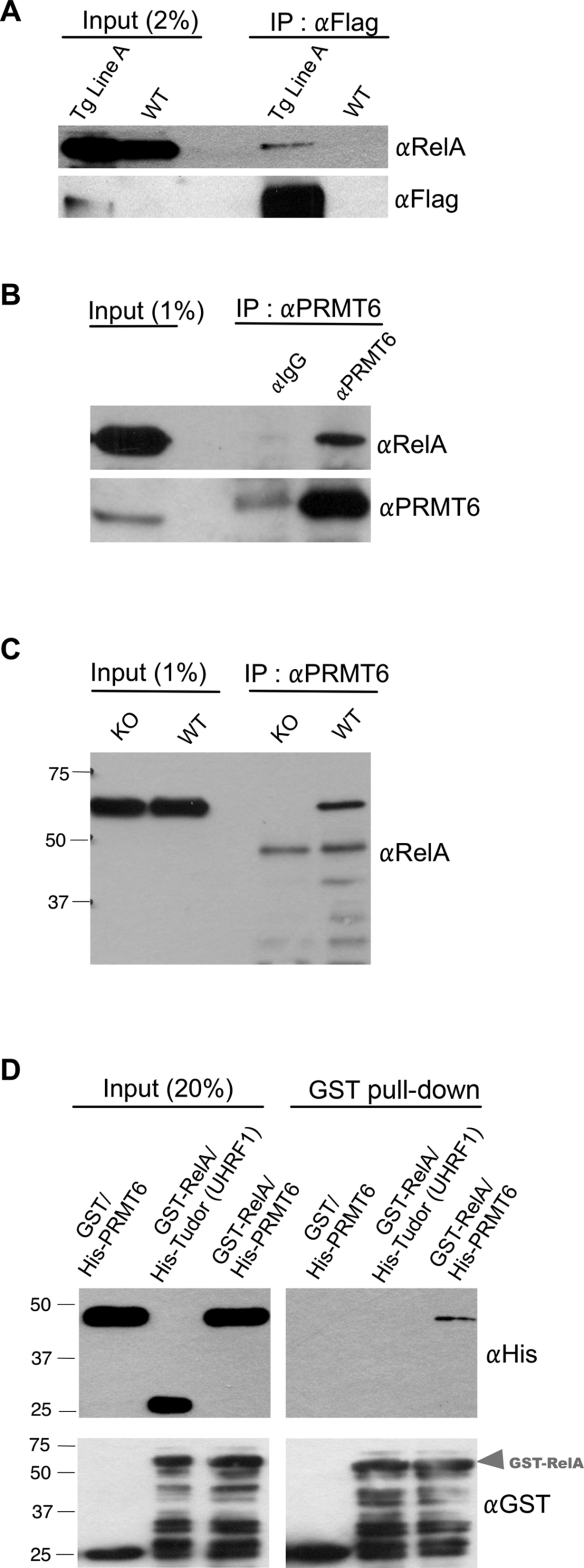
RelA coimmunoprecipitates with endogenous PRMT6 and they directly interact *in vitro*. (**A**) Primary MEFs were isolated from WT and ER*-PRMT6 Line A mice, cultured for 7 days and treated with OHT (2 μM) for 24 h. Cells were then lysed and an IP was performed using αFlag agarose. A WB using αRelA antibody reveals an interaction between the ER*-PRMT6 and the endogenous RelA in the lysate from the transgenic cells. (**B**) HEK 293 cells were grown to 90% confluency, then washed with PBS and lysed in co-IP buffer. Lysates were divided in equal volumes and incubated with αIgG or αPRMT6 antibodies overnight and with protein A/G agarose thereafter. A WB using αRelA antibody reveals an interaction between PRMT6 and RelA. (**C**) Immortalized WT and PRMT6-KO MEFs were grown to 90% confluency, and then washed with PBS and lysed in co-IP buffer. Lysates were incubated with αPRMT6 antibody (which recognized mouse PRMT6) overnight and with protein A/G agarose thereafter. A WB using αRelA antibody reveals an interaction between PRMT6 and RelA in the WT cells. (**D**) Direct binding between PRMT6 and RelA. GST and GST-RelA (1–431) were incubated with His-PRMT6 and a GST pull-down was performed. For an additional negative control, GST-RelA was also incubated with a His-Tudor-UHRF1. A WB using αHis antibody reveals direct binding between GST-RelA and His-PRMT6.

The NF-κB is known to regulate the expression of inflammatory cytokines (36). The PRMT family members CARM1 and PRMT1 have been shown to coactivate NF-κB-dependent gene expression (20,22). Thus, we asked if PRMT6 might exhibit this function as well, which would explain the elevated levels of IL-6 observed in the Line A transgenic mice. We first asked if PRMT6 is in the NF-κB complex, by performing an IP using lysates of primary MEFs from WT and Tg Line A mice using an anti-Flag agarose. Using this approach, we could co-IP endogenous RelA from the transgenic, but not the WT cells (Figure 3A). In order to exclude the possibility that the ER* portion of the fusion protein could mediate this interaction, we transiently transfected HEK 293 cells with constructs expressing GFP, GFP-CARM1 or GFP-PRMT6 along with a pCDNA-RelA vector for 48 h, and then treated the cells with TNF-α for 30 min. IP using αGFP or αIgG antibodies was performed on cell lysates. WB using αRelA antibody shows that RelA binds to GFP-CARM1 and GFP-PRMT6, but not to GFP alone (Supplementary Figure S3A). CARM1 has previously been reported to interact with RelA, and it serves as a positive control in this experiment (20). A strong interaction was also confirmed by transiently transfecting a Flag-PRMT6 (not fused to ER*) construct in HEK 293 cells followed by co-IP experiments (Supplementary Figure S3B). To further validate our data in an endogenous setting, we performed co-IP experiments and found that endogenous PRMT6 interacts with endogenous RelA in HEK 293 cells (Figure 3B). Additional co-IP experiments performed in PRMT6 WT and KO MEFs confirm the endogenous interaction between PRMT6 and RelA, and also verifies the specificity of the αPRMT6 antibody (Figure 3C).

The coactivators histone acetyltransferases p300 and its homolog, the CREB-binding protein (CBP) are known to impact NF-κB-dependent gene expression and have been shown to interact directly with RelA (37). Moreover, NF-κB-dependent gene expression involves another class of transcriptional coactivators, the steroid receptor coactivators (SRCs), which interacts with the p50 subunit of NF-κB (38). PRMT6 has recently been shown to function as coactivator of several nuclear receptors (i.e. progesterone, glucocorticoid and estrogen receptors) and to bind to SRC-1 in a mammalian two-hybrid assay (11). Therefore, we questioned if PRMT6 interacts directly with RelA or if the binding is bridged by other factors, such as SRC-1. For this purpose, GST and GST-RelA (1–431) were incubated with His-PRMT6, and a GST pull-down experiment was performed. After extensive washes, bound proteins were resolved on SDS-PAGE, followed by western blot analysis using αHis antibody. As a negative control, a His-Tudor domain (UHRF1) was incubated with GST-RelA. As shown in Figure 3D, PRMT6 and RelA interact directly in this *in vitro* assay.

### PRMT6 is a coactivator of NF-κB and its enzymatic activity is required for this function

**Figure 4.**
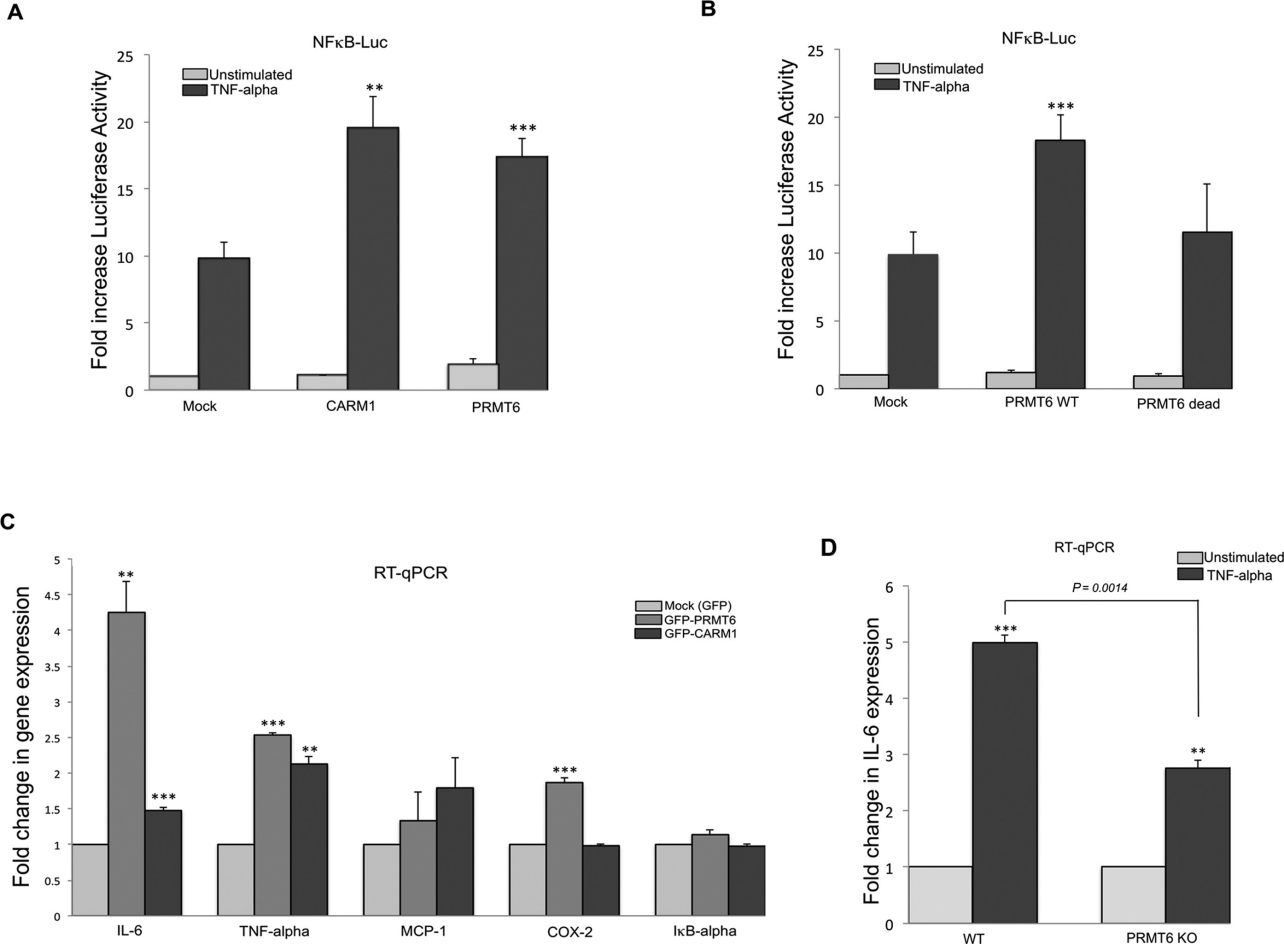
PRMT6 is a NF-κB coactivator. (**A**) HEK 293 cells were transiently cotransfected with a NF-κB firefly luciferase reporter plasmid and a Renilla luciferase construct along with a GFP vector (Mock), a GFP-CARM1 or a GFP-PRMT6 construct. Cells were left unstimulated or stimulated 20 h after transfection with TNF-α (10 ng/ml, 6 h). Error bars represent standard deviation calculated from quadruplicate luciferase assays. Mean values are expressed as fold changes in luciferase activity with the unstimulated Mock arbitrarily set as 1. The *P* values obtained for PRMT6 and CARM1 transfections compared to the Mock are 0.00016 and 0.0012, respectively. These data are representative of four independent experiments. (**B**) HEK 293 cells were transiently cotransfected with a NF-κB luciferase reporter plasmid and a Renilla luciferase construct along with an empty vector or pMyc-PRMT6 or a PRMT6 catalytic inactive form (dead). Cells were unstimulated or stimulated 20 h after transfection with TNF-α (10 ng/ml, 6 h). Error bars represent standard deviation calculated from quadruplicate luciferase assays. Mean values are expressed as fold changes in luciferase activity with the unstimulated Mock arbitrarily set as 1. The *P* values obtained for the TNF-α-treated groups of PRMT6 WT and dead enzymes are 0.0006 and 0.43, respectively, when compared to the Mock. (**C**) HeLa cells were transfected with GFP (Mock), GFP-PRMT6 or GFP-CARM1 constructs for 24 h and then stimulated with TNF-α (10 ng/ml, 60 min). Total RNA was analyzed by quantitative RT-PCR for the expression of the indicated genes and normalized against GAPDH. For each gene, the TNF-α-stimulated Mock group mean was arbitrarily set as 1. Error bars represent standard deviation calculated from triplicates. For *IL-6*, the *P* values of GFP-PRMT6 and GFP-CARM1 versus Mock are 0.005 and 0.0004, respectively. For *TNF-α*, the *P* values of GFP-PRMT6 and GFP-CARM1 versus Mock are 9.2E-07 and 0.002 respectively. For *MCP1*, the *P* values of GFP-PRMT6 and GFP-CARM1 versus Mock are 0.3 and 0.07, respectively. For *COX-2*, the *P* values of GFP-PRMT6 and GFP-CARM1 versus Mock are 5.8E-05 and 0.64, respectively. (**D**) WT and PRMT6-KO immortalized Mefs were grown to 90% confluency, at which point cells were unstimulated or stimulated with TNF-α (10 ng/ml, 60 min). Total RNA was isolated and analyzed by quantitative RT-PCR for the expression of the IL-6 gene and normalized against GAPDH. Error bars represent standard deviation calculated from triplicates. The mean value for the unstimulated groups was arbitrarily set as 1. The *P* values of the TNF-α-stimulated groups compared to the unstimulated are 0.0002 and 0.0024 for the WT and KO cells, respectively. The *P* value of the fold decrease difference for KO versus WT cells is 0.0014.

In order to test if PRMT6 functions as a coactivator of NF-κB, we first performed a luciferase assay using HEK 293 cells. PRMT6 overexpression increases the luciferase activity of a NFκB-Luc luciferase reporter plasmid (Figure 4A). We used CARM1, a known coactivator for NF-κB (20), as a positive control for this experiment. Furthermore, the enzymatic activity of PRMT6 is required for its coactivator function (Figure 4B). This was established by transfecting HEK 293 cells with catalytic active and inactive forms of PRMT6 (PRMT6 dead), and performing a NFκB-Luc luciferase assay. The inactive form of PRMT6 (VLD-KLA) has previously been used in a similar assay (39).

To further confirm that PRMT6 functions as a coactivator of NFκB, we performed quantitative RT-PCR experiments, in which we asked if, by overexpressing PRMT6 in HeLa cells, the transcription of IL-6 increases upon TNF-α stimulation. We transfected HeLa cells with constructs expressing GFP, GFP-PRMT6 or GFP-CARM1 (as positive control), and we observed enhanced transcription of IL-6 when either PRMT6 or CARM1 was overexpressed (Figure 4C). We also explored the expression levels of several other genes known to be activated in HeLa cells upon TNF-α stimulation. These included *TNF-α*, *Monocyte chemotactic protein 1* (*MCP-1/CCL-2*), *Cyclooxygenase 2* (*COX-2*) and *IκB-α* (40). Among these genes, we found that PRMT6 coactivates *TNF-α* and *COX-2* (Figure 4C). Furthermore, taking advantage of the availability of PRMT6-KO MEFs, we asked if the absence of PRMT6 might result in diminished expression of NF-κB-regulated genes. In this experiment, we clearly saw significant reduction in *IL-6* transcription in cells lacking PRMT6 (Figure 4D).

### PRMT6 causes RelA nuclear shuttling and associates with a subset of NF-κB target promoters

**Figure 5.**
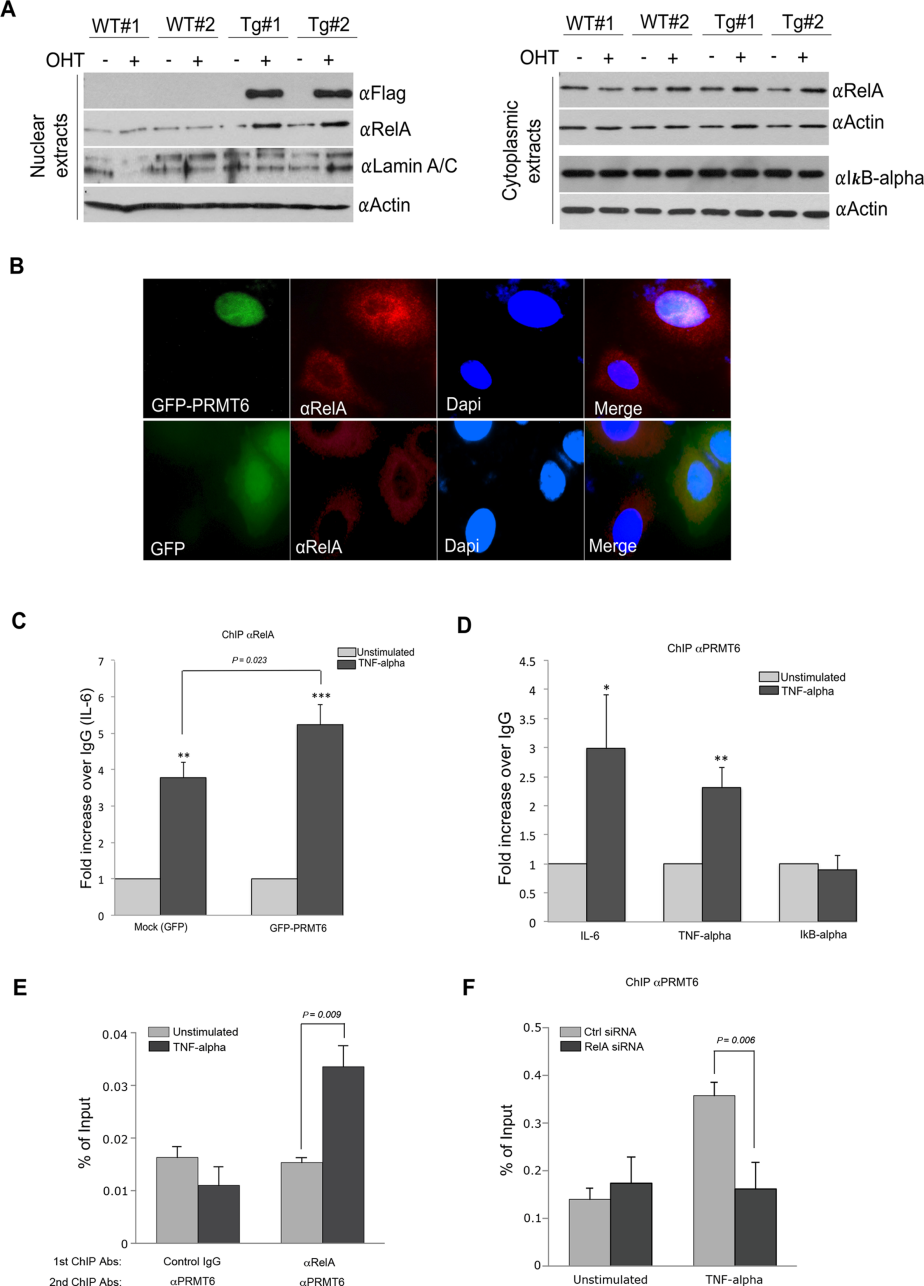
PRMT6 promotes RelA shuttling into the nucleus and ChIPs at a subset of NF-κB-regulated promoters. (**A**) Primary MEFs were isolated from WT and ER*-PRMT6 mice, cultured for 3 days and treated with OHT for 2 weeks. Cell fractionation was performed. Western analysis for the Flag-tag shows stabilization of ER-PRMT6 in the nuclear fraction, corresponding to increased nuclear RelA. WB for RelA and IκB-α on the cytoplasmic extracts shows no change in the levels of these two proteins. (**B**) Immunofluorescence using a αRelA antibody following transient transfection of GFP-PRMT6 in HeLa cells shows increased nuclear RelA in transfected cells. A GFP-expressing construct was used as negative control. (**C**) HeLa cells were transiently transfected with a GFP or GFP-PRMT6 expressing vector for 24 h, at which time cells were left untreated or treated with TNF-α (10 ng/ml, 30 min). ChIP analyses at the κB consensus region of *IL-6* promoter were then performed using αRelA antibody. Values were normalized to an intergenic region 8000 bp upstream *IL-6* consensus sequence. For each transfection, the mean value of the unstimulated group was arbitrarily set as 1. Error bars represent standard deviations calculated from triplicates. The *P* values of the TNF-α-stimulated groups compared to the unstimulated are 0.0024 and 0.0003 for the Mock and GFP-PRMT6-transfected cells, respectively. The *P* value of the fold increase difference for PRMT6-overexpressing cells versus the Mock is 0.023. (**D**) HeLa cells were grown to 90% confluency, at which point cells were left untreated or treated with TNF-α (10 ng/ml, 30 min). ChIP analyses at the κB consensus region of *IL-6*, *TNF-α* and *IκB-α* promoters were then performed using αPRMT6 antibody. Values were normalized to an intergenic region 8000 bp upstream *IL-6* consensus sequence. Error bars represent standard deviations calculated from triplicates. The mean value of the unstimulated groups was arbitrarily set as 1. The *P* values of the TNF-α-stimulated groups compared to the unstimulated are 0.024, 0.0053 and 0.84 for *IL-6*, *TNF-α* and *IκB-α* genes, respectively. (**E**) The recruitment of PRMT6 and RelA to the IL-6 promoter consensus region was evaluated by ChIP/re-ChIP experiment. HeLa cells were either left unstimulated or treated with TNF-α (10 ng/ml, 30 min) before the ChIP/re-ChIP experiments were performed using indicated antibodies. The ChIP DNA was analyzed by qPCR with primers for the IL-6 consensus region. (**F**) ChIP experiment was performed using αPRMT6 antibody in control and RelA-knockdown HeLa cells, which were either left unstimulated or treated with TNF-α.

Having established that PRMT6 coactivates NF-κB, we investigated the possible mechanism for this function. First, we explored the possibility that, when overexpressed, PRMT6 may promote the movement of NF-κB into the nucleus. For this purpose, we isolated primary MEFs from WT and ER*-PRMT6 embryos and treated them with OHT for 2 weeks. Cells were then fractionated and subjected to western blot analysis using αRelA antibody. We observed an increase of nuclear RelA upon the Tamox-induced stabilization of ER*-PRMT6 (Figure 5A). This effect on RelA nuclear shuttling was seen in MEFs isolated from additional embryos (data not shown). No change in global levels of RelA was observed in primary MEFs (Supplementary Figure S4), and no alterations in IκB-α levels were detected (Figure 5A). In order to confirm this phenomenon, we performed IF in HeLa cells upon transient transfection with a GFP-PRMT6 construct. A GFP-expressing construct was transfected in parallel for negative control. As shown in Figure 5B, cells overexpressing PRMT6 (which localizes strongly to the nucleus) show increased RelA in the nucleus. Thus, the overexpression of both GFP-PRMT6 and ER*-PRMT6 causes increased nuclear RelA. This established that the ER* component of the ER*-PRMT6 fusion is not responsible for this translocation of RelA, but rather it is the PRMT6 protein itself.

We then asked if the promotion of nuclear shuttling of RelA upon PRMT6 overexpression results in increased localization of RelA at target promoters. We transfected HeLa cells with either GFP or GFP-PRMT6, and looked at RelA occupancy of the *IL-6* promoter after TNF-α stimulation. In PRMT6-overexpressing cells, RelA recruitment to the *IL-6* promoter is significantly enhanced (Figure 5C). Next, we asked if the absence of PRMT6 might impair NF-κB nuclear shuttling. We utilized an immortalized PRMT6-KO MEF line along with WT MEFs and performed IF analyses as well as ChIP analyses at the *IL-6* promoter using an αRelA antibody. Loss of PRMT6 does not impede RelA nuclear translocation (Supplementary Figure S5A) or recruitment to the *IL-6* gene promoter (Supplementary Figure S5B).

Posttranslational modification of RelA by PRMT6 could account for its increased nuclear localization. We, thus, tested the possibility that PRMT6 may methylate RelA by performing *in vitro* methylation assays. In this *in vitro* methylation assay, we observed robust methylation of histone H3, as well as automethylation of PRMT6 itself, but no methylation of recombinant RelA (Supplementary Figure S6). Thus, we questioned if PRMT6, like CARM1 (20), might instead be recruited at selective κB target promoters that are upregulated upon TNF-α stimulation (Figure 4C). To determine this, we performed ChIP experiments at the promoter region of the *IL-6, TNF-α* and *IκB-α* genes in HeLa cells using an αPRMT6 antibody. As expected, we observed enrichment of PRMT6 at the transcriptional start site of *IL-6* and *TNF-α*, but not *IκB-α* (Figure 5D). By performing a re-ChIP experiment, we could show that RelA and PRMT6 reside together at the IL-6 promoter (Figure 5E). Knockdown of RelA reduces its recruitment to the IL-6 promoter (Supplementary Figure S5C and D), as would be expected. Importantly, under these same RelA knockdown conditions, PRMT6 is not recruited to the IL-6 promoter (Figure 5F). These data strongly suggest that PRMT6 is recruited to chromatin at selective NF-κB target promoters, through a direct interaction with RelA, where it helps promote transcription.

## DISCUSSION

Chromatin is an active platform that integrates external and internal cellular signals into dynamic changes in gene expression. Studies on high-order complexes during activation of NF-κB-regulated genes and the consequent impact on chromatin remodeling through histone posttranslational modification have increased enormously, and it has become clear that the NF-κB transactivation is far more complex than anticipated (41). A number of coactivators and mediators have been discovered to regulate its transcriptional activity; indeed, NF-κB recruits a coactivator complex that has striking similarities to that recruited by steroid nuclear receptors and the role of factors that impact histone tail posttranslational modifications has become evident (37). Histone arginine methylation by protein arginine methyltransferases has elicited a great deal of interest due to increasing evidence that it regulates chromatin remodeling and gene expression by providing docking sites for specific readers or effector molecules (13,42,43).

In the current study, we have shown that PRMT6 functions as a coactivator for NF-κB-mediated gene transcriptional activity. We arrived at this finding by generating a transgenic mouse model that overexpresses PRMT6, and observed an activated inflammatory response in these mice. The specific involvement of PRMT6 in upregulating inflammatory genes was confirmed by demonstrating that overexpression of PRMT6 enhances NF-κB transcriptional activity in cultured cell-based luciferase assays and quantitative RT-PCR experiments. The activity of PRMT6 was clearly necessary for the coactivator function. ChIP analysis demonstrated that PRMT6 was recruited to the IL-6 promoter upon TNF-α stimulation. Moreover, overexpression of PRMT6 caused RelA shuttling into the nucleus, which could justify, at least in part, the mechanism underlying its coactivator function. As a consequence of enhanced nuclear translocation of RelA, we observed increased RelA localization at target promoters. The absence of PRMT6 did not impair RelA nuclear shuttling or accumulation at target promoters. However, the reduction of RelA levels by a knockdown approach did impair PRMT6 accumulation at target promoters. Notably, PRMT6 has been found overexpressed in many types of human cancers (2,19,44); therefore, the facilitation of NF-κB nuclear shuttling could be an important mechanism contributing to NF-κB-dependent gene activation in cancer (45). Cells lacking PRMT6 displayed reduced transcription of the *IL-6* gene, strongly supporting a role for PRMT6 as a NF-κB coactivator.

One question remains to be answered: what is the role of arginine methylation in PRMT6 coactivation of NF-κB transcription? A number of posttranslational modifications (PTMs) on NF-κB subunits have been shown to affect its stability, function, subcellular localization and binding to DNA (46). It has recently been published that PRMT5 (a type-II arginine methyltransferase) methylates p65/RelA (23). This methylation event appears to increase the ability of NF-κB to bind to κB elements and to drive gene expression. We investigated the possibility of PRMT6 methylating RelA using an *in vitro* methylation assay, but we found that this is not the case. Other modes of PRMT6 regulation could be through the methylation of non-histone proteins that are associated with the NF-κB complex or through its ability to methylate histones. Both these regulatory mechanisms are currently under investigation by our group.

It has been shown that the mode of action of PRMT6 is similar to PRMT1 and CARM1, in that it functions as a secondary coactivator to p160/SRC proteins (11). PRMT6 binds to the AD2 domain of SRC-1, and functions synergistically with SRC-1 to coactivate ERα. Therefore, PRMT6 can be added to the list of steroid hormone receptor coactivators that are able to interact with SRC-1 to enhance transcriptional activation. The likely mechanism by which PRMT6 will function as a coactivator has recently come to light with the finding that it methylates a site in the core of histone H3, H3R42me2a (12). Importantly, the side chain of this arginine residue interacts with the DNA minor groove and methylation of this site is proposed to sterically interfere with this interaction, as well as remove a potential hydrogen bond donor, thus destabilizing the histone:DNA interaction. Indeed, the incorporation of semi-synthetic H3R42me2a into core histone octamers, and use of these octamers in reconstituted chromatin for the generation of chromatinized transcription template revealed this to be the case. Unfortunately, there are currently no antibodies available to the H3R42me2a mark, so we are unable to test the hypothesis that this histone methylation site is responsible for the coactivator functions of PRMT6 in the context of nuclear receptor and NF-κB signaling.

This is the first gain-of-function PRMT mouse model to be described. The phenotype of this mouse is obviously complex, with activated PRMT6 likely impacting the methylation levels of many substrates. The impacted substrates include dramatic changes in the histone code at the H3R2me2a site (which we have observed), and possibly other arginine residues on histones, as well as elevated methylation levels on non-histone substrates. In the future, this type of mouse model will be valuable for the *in vivo* analysis of small molecule PRMT inhibitors that are currently being developed both in academic and pharma settings (47). Ideally, good PRMT6-specific small molecule inhibitors would allow the survival of Tamox-treated ER*-PRMT6 transgenic mice.

## SUPPLEMENTARY DATA

Supplementary Data are available at NAR Online.

SUPPLEMENTARY DATA
