# Supplementary material for: A gain-of-function mouse model identifies PRMT6 as a NF-κB coactivator
Source: Nucleic Acids Res. 2014 Jun 17;42(13):8297–309. doi: 10.1093/nar/gku530 (PMC4117762; doi:10.1093/nar/gku530)
Supplement: SUPPLEMENTARY DATA [file supp_42_13_8297__index.html]

A gain-of-function mouse model identifies PRMT6 as a NF-κB coactivator — SUPPLEMENTARY DATA 

# A gain-of-function mouse model identifies PRMT6 as a NF-κB coactivator

## SUPPLEMENTARY DATA

**Files in this Data Supplement:**

- SUPPLEMENTARY DATA
