# Supplementary material for: A gain-of-function mouse model identifies PRMT6 as a NF-κB coactivator
Source: Nucleic Acids Res. 2014 Jun 17;42(13):8297–309. doi: 10.1093/nar/gku530 (PMC4117762; doi:10.1093/nar/gku530)
Supplement: SUPPLEMENTARY DATA [file supp_gku530_nar-00440-2014-File007.docx]

**SUPPLEMENTAL DATA**

**Supplementary methods**

**In Vitro Methylation assay**

In vitro methylation reactions were carried out in 30 μl of phosphate-buffered saline (pH = 7.4.) containing 0.5–1.0 μg of substrate, 3 μg of recombinant GST-PRMT6 and 0.42 μm [^3^H]S-adenosyl-l-[methyl-^3^H]methionine (79 Ci/mmol from a 7.5 μm stock solution; PerkinElmer Life Sciences). The reaction was incubated at 30 °C for 1 h and then separated on SDS-PAGE, transferred to a PVDF membrane, treated with En^3^Hance™ (PerkinElmer Life Sciences), and exposed to film for 1–3 days at –80 °C.

**Isolation and culture of mouse primary keratinocytes**

Briefly, 1-2 day old pups were sacrificed according to standard procedures and skin was removed. Skins were placed on Trypsin-EDTA O/N 4 °C, then epidermis was carefully removed from the dermis, chopped with sterile blades and transferred in 50-mL falcon tube containing Super media (with pen-strep, 10% FBS). Cells were then strained through a 70 μm strainer after mixing 5 times with a 10 mL sterile pipette. Cells were left to adhere to a petri dish for 3 hrs in Super media, then the media was replaced with keratinocyte basal media (Lonza, cat.# CC-3158) containing FBS 10%, pen-strep and additioned with components of the Lonza Kit KGM-2 SingleQuots (Cat.# CC-4152).

**Knockdown of RELA expression by siRNA**

Hela cells were transfected with 30 nM of ON-TARGETplus SMART pool siRNA targeting human RELA (Cat# L003533-00, GE Healthcare). Both western blot and ChIP assay were performed 48 hours after transfection.

**FIGURE LEGENDS**

**Figure S1. Line A mice show nuclear translocation and stabilization of ER*-PRMT6 in the liver upon Tamox injection.** Immunohistochemistry (IHC) using an αFlag antibody shows that upon IP injection of 1 mg Tamox (5 days) nuclear translocation of ER*-PRMT6 is induced in the liver of Line A mice. The vehicle (sunflower oil) was injected as a control.

**Figure S2. Primary keratinocytes from ER*-PRMT6 mice display increased transcription of IL-6.** Primary keratinocytes were isolated from 12 newborn mice (6 WTs and 6 Tgs). Pups were taken from two different litters. All cells were cultured for two days, treated with OHT (1μM) for 48 hrs, and then treated with TNF-α for 60 min, at which point and RNA isolation and cell lysates were extracted. **A**, Western analysis for the flag-tag on cell lysates shows stabilization of the ER*-PRMT6 in the Tg mice. Western analysis using an αkeratin-5 antibody shows that these are indeed keratinocytes (as negative control we loaded a cytoplasmic extract of Line A MEFs). **B**, RNA was analyzed by quantitative RT-PCR for the expression of IL-6 and normalized for GAPDH. The RQ difference between the two groups of isolated keratinocytes is statistically significant (*p* = 0.0335).

**Figure S3. RelA co-immunoprecipitates with GFP-PRMT6 and Flag-PRMT6.** (**A**) HEK 293 cells were transiently co-transfected with a pcDNA-RelA vector and constructs expressing GFP, GFP-CARM1 or GFP-PRMT6 for 48 hrs, then treated with TNF-alpha for 30 min. Immunopreciptation using αGFP or αIgG antibodies was performed on the cell lysates. A Western blotting using αRelA antibody shows that RelA binds to GFP-CARM1 and GFP-PRMT6, but not to GFP alone. (**B**) HEK 293 cells were transiently co-transfected with a pCDNA-RelA vector and a pCAGGS-Flag-PRMT6 construct for 48 hrs, and then treated with TNF-α for 30 min. IP using αFlag agarose was performed on the cell lysates. A Western analysis using αRelA antibody shows that RelA is co-IPed from the cells transfected with Flag-PRMT6, but not from the Mock (cells transfected with pCDNA-RelA vector and empty pCAGGS vector).

**Figure S4. Global levels of RelA do not increase upon PRMT6 overexpression.** Primary MEFs were isolated from two WT and two ER*-PRMT6 mice, cultured for 3 days and treated with OHT (1μM) for 3 days. Total cell lysates were analyzed by Western using αRelA and αFlag antibodies. Cells that display robust expression of ER*-PRMT6 do not show increased total levels of RelA.

**Figure S5. PRMT6 is not essential for a correct RelA nuclear localization upon stimulus**. (**A**) IF using αRelA antibody on un-stimulated and TNF-alpha stimulated WT and PRMT6 KO Mefs is shown here. (**B**) WT and PRMT6 KO Mefs were grown to 80-90% confluency, at which point cells were left untreated or treated with TNF-alpha (10ng/mL, 30 minutes). ChIP analyses at the κB consensus region of IL-6 promoter were then performed using αRelA antibody. Values were normalized to an inter-genic region 8000 bp upstream *IL-6* consensus sequence. Error bars represent standard deviations calculated from triplicates. The mean value of the un-stimulated groups was arbitrarily set as 1. The *p* values of the TNF-alpha-stimulated groups compared to the un-stimulated are 0.0008 and 0.0003 for the WT and KO cells, respectively. The Fold difference between WT and KO stimulated groups is not statistically significant (*p*= 0.15). The absence of PRMT6 in the KO cells was confirmed by Western analysis using an αPRMT6 antibody (inset). (**C**) Hela cells were transfected with control siRNA or siRNA targeting RelA. RelA knockdown efficiency was detected by Western blotting using αRelA, αPRMT6 and αActin antibodies. (**D**) ChIP experiment was performed using αRelA antibody in control and RelA knockdown Hela cells, which were either left unstimulated or treated with TNF-alpha (10ng/mL, 30 minutes). The ChIP DNA was analyzed by qPCR with primers for the IL-6 consensus region.

**Figure S6. PRMT6 does not methylate RelA *in vitro*.** GST-PRMT6 was incubated with recombinant histone H3, GST, or GST-RelA (1-431) and the methyl-donor, tritiated S-Adenosyl-methionine (AdoMet), for 30 min. The reactions were then resolved by SDS-PAGE and transferred to a PVDF membrane. A fluorography revealed strong activity of PRMT6 on recombinant histone H3, while no activity was detected on RelA (1-431).

**Figure S1**

**
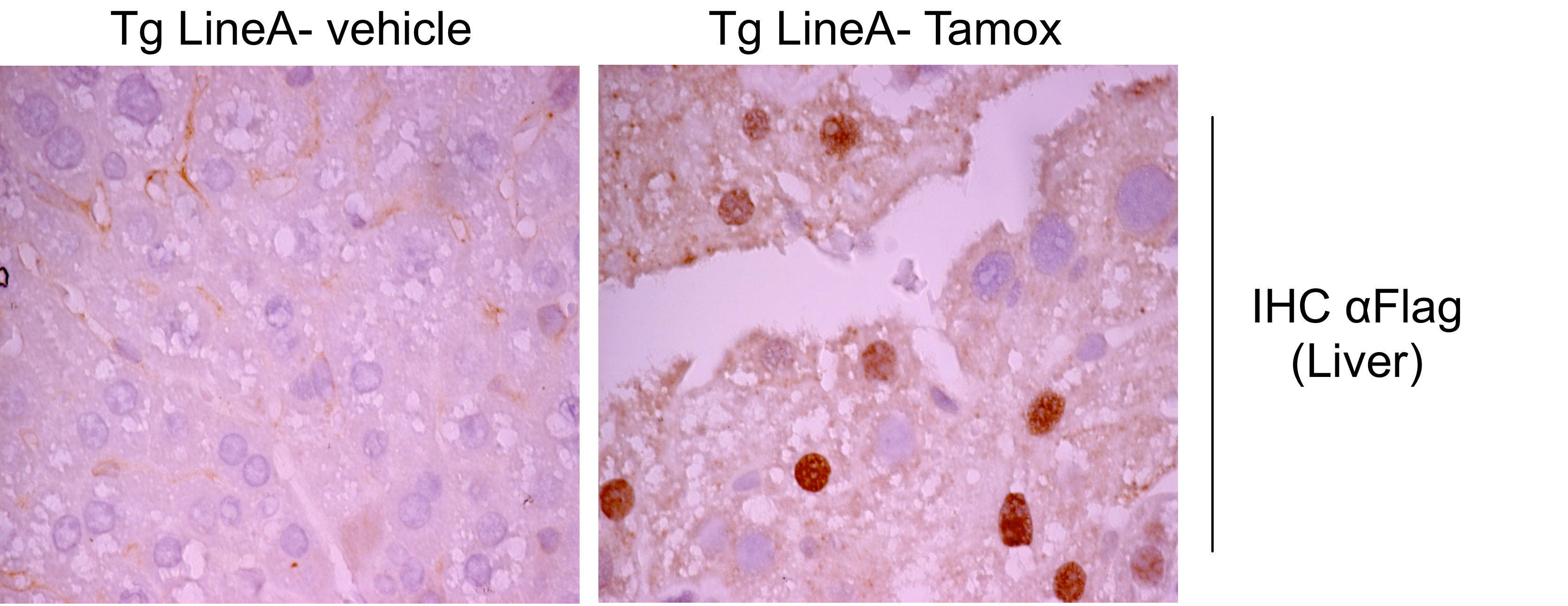
Figure S2**

**
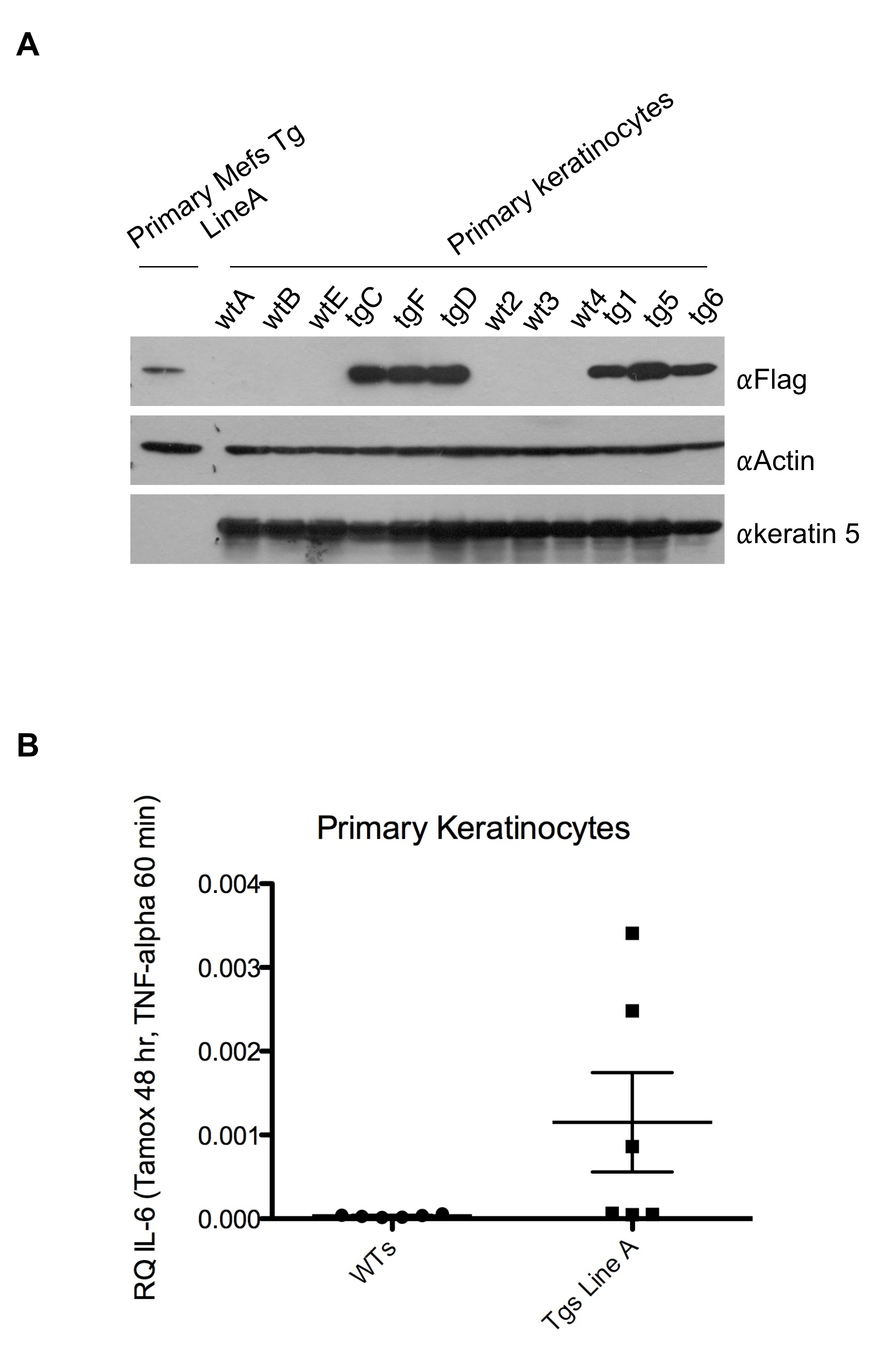
Figure S3**

**
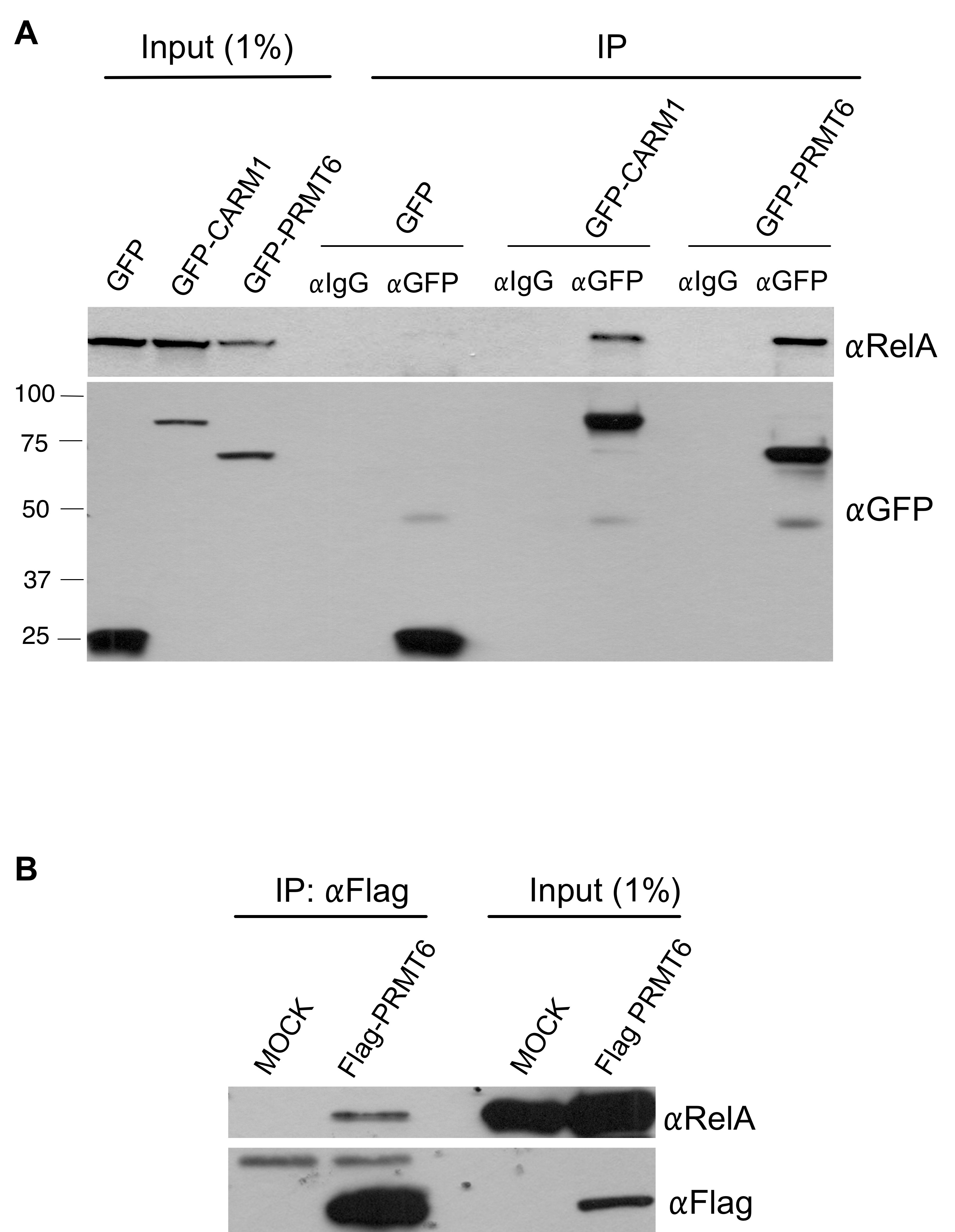
Figure S4**

**
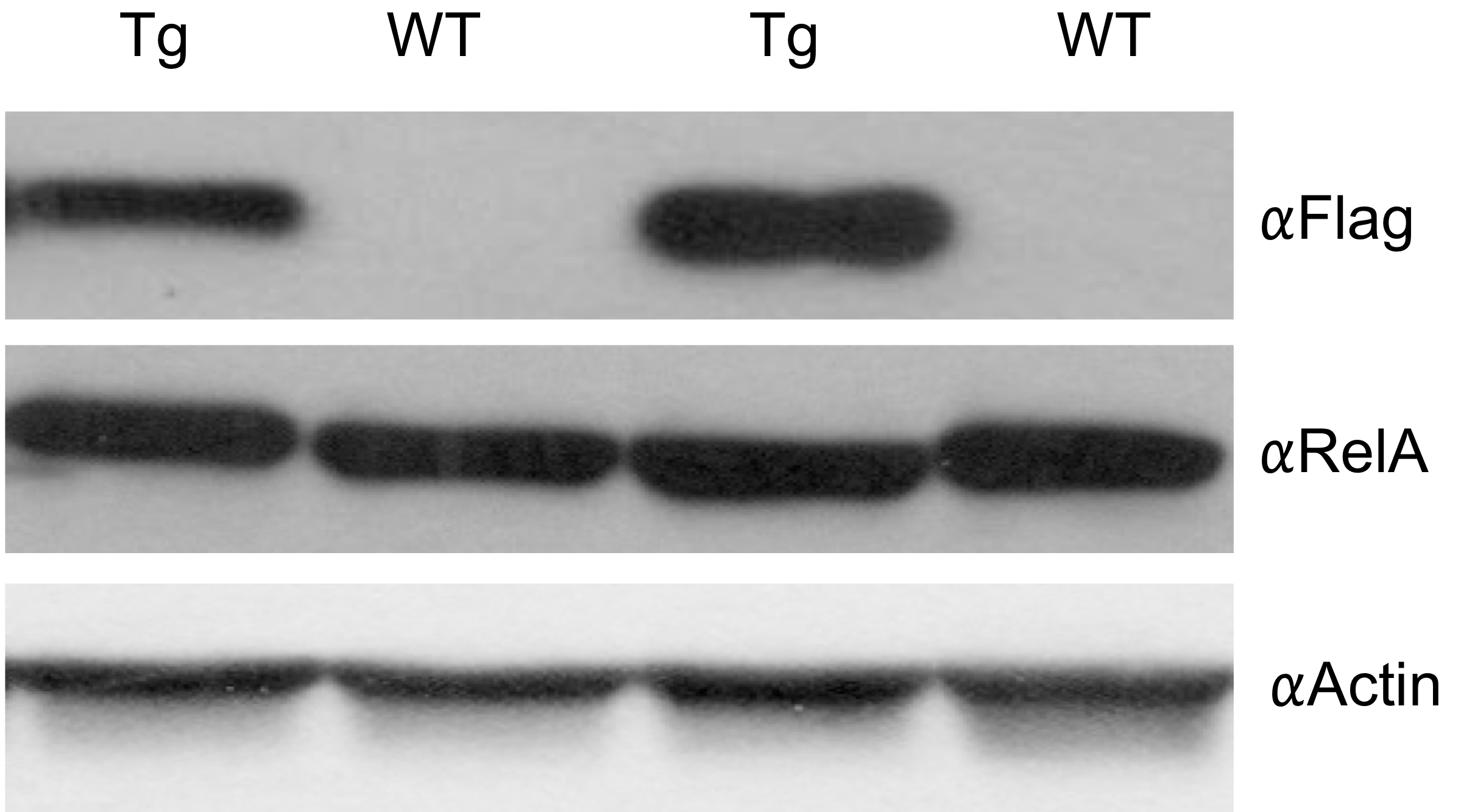
Figure S5**

**
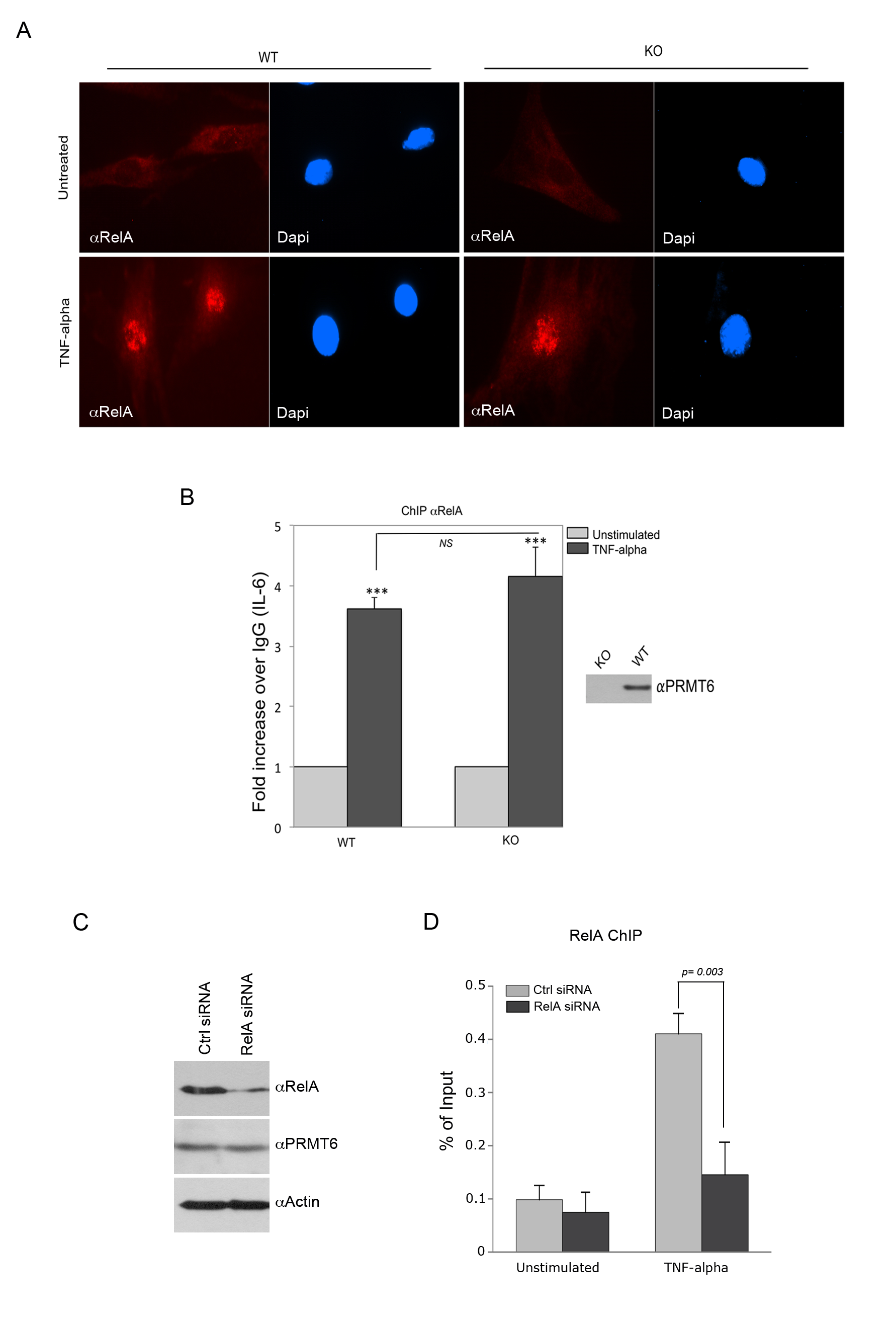
**

**
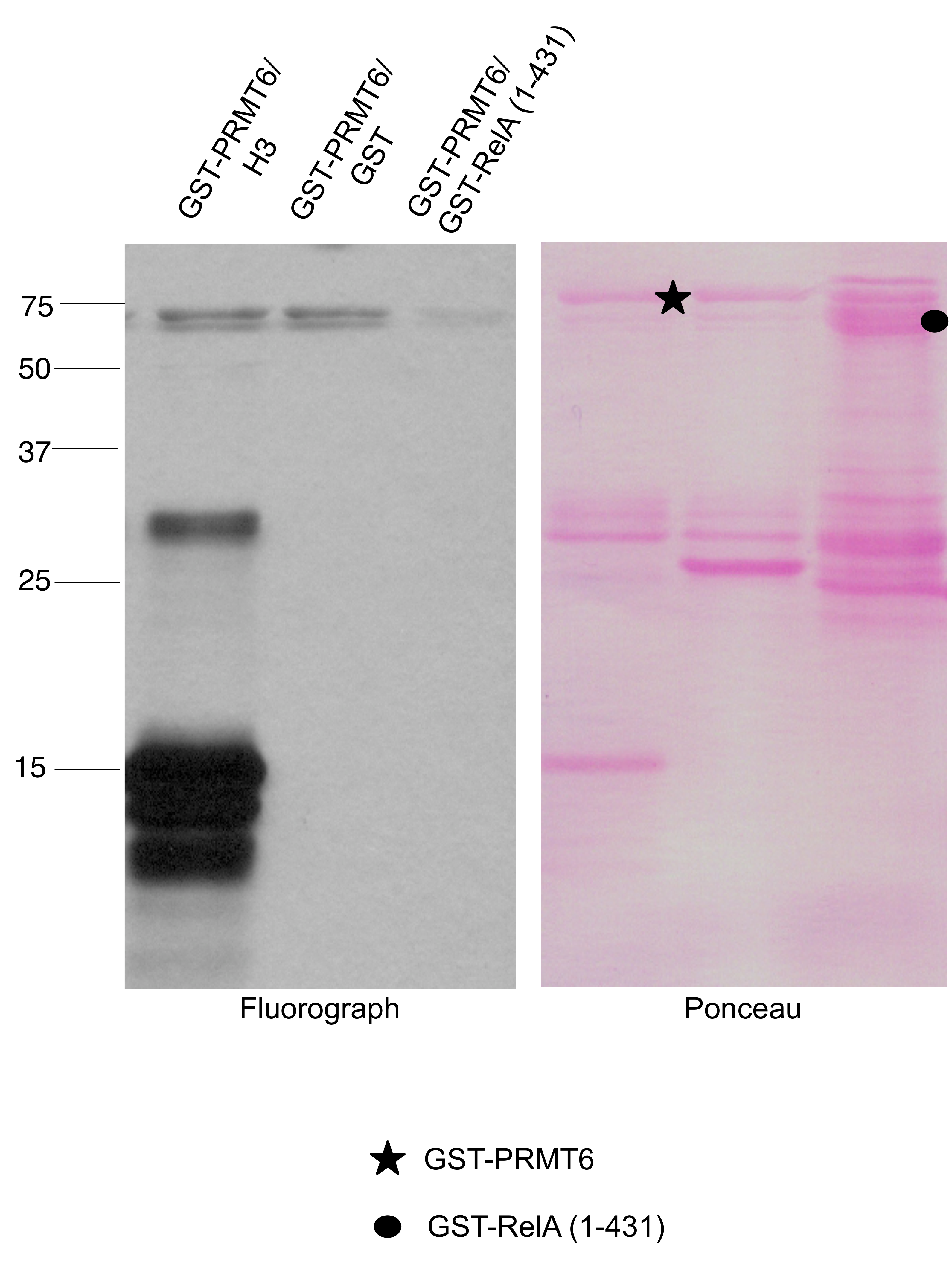
Figure S6**
